# An Electronic Health Tool to Prepare for the First Orthopedic Consultation: Use and Usability Study

**DOI:** 10.2196/13577

**Published:** 2019-11-28

**Authors:** Aniek A O M Claassen, Thea P M Vliet Vlieland, Vincent J J F Busch, Henk J Schers, Frank H J van den Hoogen, Cornelia H M van den Ende

**Affiliations:** 1 Department of Rheumatology Sint Maartenskliniek Nijmegen Netherlands; 2 Department of Orthopaedics, Rehabilitation and Physical Therapy Leiden University Medical Center Leiden Netherlands; 3 Department of Orthopaedic Surgery Sint Maartenskliniek Nijmegen Netherlands; 4 Radboud Institute for Health Sciences, Department of Primary and Community Care Radboud University Medical Center Nijmegen Netherlands; 5 Department of Rheumatology Radboud University Medical Center Nijmegen Netherlands

**Keywords:** osteoarthritis, patient education, consultation, eHealth, smartphone, use

## Abstract

**Background:**

The use of electronic health (eHealth) technology to prepare patients with hip or knee osteoarthritis (OA) for their first orthopedic consultation seems promising. Exploration of the use and usability of an educational eHealth tool may highlight potential modifications that could increase patient engagement and effectiveness.

**Objective:**

This study aimed to (1) identify the use and usability of a stand-alone educational eHealth tool for patients with suspected hip or knee OA, (2) explore whether the recorded questions in the eHealth tool were in line with an existing widely used question prompt list, and (3) investigate whether user characteristics are related to use and usability.

**Methods:**

We used data from 144 participants in the intervention group of a randomized controlled trial, who were asked to use the educational eHealth tool to prepare for their upcoming first orthopedic consultation. We defined users and nonusers based on whether they had opened the tool at least once. Users were characterized as active or superficial depending on the extent of their use of the tool. The recorded questions for the consultation preparation were categorized into themes fitting 3 predefined questions or in a remaining category. Usability was measured using the System Usability Scale (SUS, 0-100). Data were collected including the patient demographic and clinical characteristics, knowledge of OA, and internet and smartphone usage in daily life. The characteristics associated with users and nonusers were analyzed using a multivariable logistic regression analysis.

**Results:**

A total of 116/144 (80.6%) participants used the educational eHealth tool, of whom 87/116 (75.0%) were active users. Of the three components of the tool (information, my consultation, and medication), medication was the least used (34%). On the basis of recorded questions of the users, the fourth predefined question could be proposed. The mean (SD) SUS score was 64.8 (16.0). No difference was found between the SUS scores of superficial and active users (mean difference 0.04, 95% CI −7.69 to 7.77). Participants with a higher baseline knowledge of OA (odds ratio [OR] 1.2, 95% CI 1.0 to 1.4) and who used the internet less frequently in their daily life (OR 0.6, 95% CI 0.5 to 0.9) were more likely to use the educational eHealth tool. We found no differences between the demographics and clinical characteristics of the superficial and active users.

**Conclusions:**

Based on the results of this study, it can be concluded that the use of an educational eHealth tool to prepare patients with hip and knee OA for the first orthopedic consultation is feasible. Our results suggest some improvements that should be made to the content of the tool to improve its usability. No clear practical implications were found to support the implementation of the educational eHealth tool in specific subgroups.

**Trial Registration:**

Netherlands Trial Register NTR6262; https://www.trialregister.nl/trial/6262

## Introduction

### Background

Osteoarthritis (OA) is an age-related, degenerative joint disease and one of the most common causes of disability around the world [1]. International guidelines recommend nonsurgical treatments, such as lifestyle education, exercise therapy, weight loss if overweight, and pain medication, as a primary approach to manage hip or knee OA in the early stages [2,3]. Once these conservative treatment options have been adequately tried and failed, or in the case of diagnostic uncertainty, a referral to an orthopedic surgeon should be considered for further diagnostic evaluation and consideration of surgical interventions, for example, a total joint replacement (TJR) [2]. Patients with hip and knee OA often expect action to be taken when referred to an orthopedic surgeon [4], in particular, the planning of a TJR; however, only one-third to a half of referred patients are eligible for a TJR [5,6]. It is therefore conceivable that patients’ expectations about the consultation may not always be met, resulting in patients being dissatisfied [7]. A solid preparation for the consultation might help to streamline patient expectations [8].

In general, educational interventions can help patients to be more prepared for a consultation by providing information on treatment options [6] and by assisting patients in reflecting on their own situation (eg, monitoring symptoms or recording medical history) [9-11]. Moreover, the use of self-prepared or provided question prompt lists for patients to ask or questions to be expected from the health care provider can facilitate the exchange of information during consultations [8,12,13]. Previous research has shown that the use of conventional educational tools to prepare patients for consultations and to aid treatment decision making in OA is associated with lower health care costs because it may postpone unnecessary early surgery [11,14].

The growing and emerging opportunities in the use of electronic health (eHealth) can be harnessed to further develop educational interventions with the potential to improve efficiency and lower costs [15]. To contribute to the emerging field of eHealth for OA and to support patients, an educational eHealth tool was developed to help hip and knee OA patients prepare for their first orthopedic consultation. This stand-alone smartphone and Web-based intervention provides information on treatment options for hip and knee OA, the option to prepare for a consultation by preparing questions, and enables patients to monitor their symptoms and medication use. A randomized evaluation of this educational eHealth tool showed that it did not influence patient satisfaction with their consultation, but it did have small effects on patient knowledge of OA and their treatment expectations (data not published yet). These results were less promising than expected; therefore, it seemed important to further explore the actual use of the intervention. Data on the usage of an intervention or its components, and its usability can provide information on potential intervention modifications that encourage engagement and, likely, effectiveness [16].

### Objectives

The aim of this study was to identify the use and usability of the aforementioned educational eHealth tool. We therefore describe the user rates of different components of the tool and explore how the preparation component of the application is used (eg, which questions do participants prepare) and whether these questions are in line with an existing widely used question prompt list [17]. Our second aim was to investigate whether certain user characteristics are related to the use and usability of the educational eHealth tool to provide points of support for its implementation.

## Methods

### Design and Setting

The data for this study were collected as part of a randomized controlled trial (RCT; Dutch Trial Register NTR6262) evaluating the effect of an educational eHealth tool compared with standard care practices, which was carried out between March 2017 and May 2018 at the outpatient department for Orthopedic Surgery at the Sint Maartenskliniek, Nijmegen, the Netherlands. Baseline and follow-up data for the intervention group and data retrieved from the backend of the educational eHealth tool were used in this study. All patients gave their informed consent for participation. The Medical Ethics Committee on Research Involving Human Subjects (CMO) Region Arnhem-Nijmegen (study number 2016-3096) waived ethical approval because it is not required for this type of study under Dutch law.

### Participants and Procedure

Patients who had a scheduled visit for a new treatment episode at the outpatient clinic of Orthopedic Surgery at the Sint Maartenskliniek, Nijmegen, were checked for their eligibility. The inclusion criteria were (1) age 18 years or above, (2) the referral letter of general practitioner or the referring specialist mentioning the (suspected) diagnosis of OA in the knee or hip, and (3) no previous visit to the department of Orthopedic Surgery at the Sint Maartenskliniek for that index joint. The exclusion criteria were (1) inability to read or understand Dutch, (2) not possessing a smartphone, computer, or tablet, or (3) not having an email address. Eligible patients were invited to participate through a letter providing information on the study. Patients who were willing to participate received further information about the study by email and were asked to fill in a baseline Web-based questionnaire 2 to 5 weeks before their consultation. Participants who were randomly assigned to the intervention group received an email with personal login details and an information flyer about the installation and use of the educational eHealth tool. The tool could be used during the 2 weeks before the scheduled consultation. One day after their consultation, the participants received a link to a follow-up Web-based questionnaire. All clinical data were collected using the electronic data capture and management program Castor EDC.

### Intervention

The educational eHealth tool was developed on the initiative of patients, and in collaboration with patients with OA and health care professionals, using an 8-step method of persuasive design [18,19] followed by an iterative process of development involving 4 cycles of development, user-testing, adaptation, retesting, and finalizing. The tool was available as a mobile app (Android and iOS) and in a Web-based version (Comaxx, digital bureau). The tool consists of 3 parts, *information, my consultations,* and *medication*, covering the following functionalities: (1) short facts and information on OA and treatment modalities, based on a stepped-care strategy for OA [20], (2) preparation for the upcoming consultation consisting of predefined questions to answer (eg, “How long do your symptoms exist?” and “Do you have morning stiffness of the joint?”), and space to record additional questions the patient would like to ask the orthopedic surgeon, (3) the option to monitor pain and fatigue during the week before the consultation (4) a list of medication used (eg, dosage), with the option to set reminders for intake, and (5) the option to create a visual timeline with the scheduled consultation, assessments, and preparation. Users could earn 3 achievement awards while using the tool: one when they had scrolled through all information parts, one when an upcoming consultation was detailed (record date, place, reason of consultation and open question list) in the educational eHealth tool, and one when medication use is registered in the tool. For further details on the development and functionalities of the eHealth tool, see Multimedia Appendix 1.

### Assessments

#### Use

On the basis of objective user data extracted from the backend of the educational eHealth tool, participants were classified as a *user* or *nonuser*. *Users* of the educational eHealth tool were defined as *opening the tool at least once*, while *nonusers* were those participants who did not open the educational eHealth tool at all. *Users* were further defined as *active* or *superficial*. If a participant had opened the tool and earned at least one achievement award, they were defined as an *active user*. Participants were defined as *superficial users* if they had used the tool but had not earned any achievements.

#### Preparation

The questions that patients recorded in the educational eHealth tool in preparation for their consultation were extracted from the backend of the tool. The questions were categorized into 3 themes, based on the 3 good preparation questions outlined by Shepherd et al: “What are my options?,” “What are the possible benefits and harms of those options?,” and “How likely are each of the benefits and harms to happen to me?” [18]. The Dutch versions of these 3 questions are implemented in several hospitals in the Netherlands. In this study, we chose to use the Dutch-implemented version, in which the last question is slightly adjusted and could better be translated as “What does this mean in my situation?” If the question did not fit 1 of the 3 themes, it was put in a *remaining* category, which was subsequently further defined based on the nature of questions assigned to that category. This categorization was performed independently by a research assistant and a researcher. Any disagreement was resolved by discussion, and if a consensus was still not reached, the third researcher was consulted.

#### Usability

In the follow-up questionnaire, the usability of the educational eHealth tool was assessed using the 10-item System Usability Scale (SUS) [21]. The items, which covered complexity, ease of use, and willingness to use the tool, among other factors, were scored on a 5-point Likert scale (*strongly disagree* to *strongly agree*). The final scores for the SUS could range from 0 to 100, where higher scores indicate better usability. The SUS is thought to be a robust, valid, and versatile questionnaire [22]. The extent to which patients were satisfied with the tool was measured by asking the patients to rate their satisfaction on a Numeric Rating Scale (NRS) ranging from 0 to 10, with higher scores indicating a higher satisfaction.

#### Demographic and Clinical Patient Characteristics

Demographic data were collected on the gender, age, body mass index (BMI), marital status, level of education, and work status of the patients. Clinical characteristics were collected on the OA location (hip or knee) and duration of symptoms (years). Western Ontario and McMaster Universities Osteoarthritis Index (WOMAC) scores were used for pain and function [23]. These scores were derived from completed Dutch Knee/Hip injury and Osteoarthritis Outcome Score questionnaires [24,25], and were presented as standardized scores (0-100), with higher scores indicating more pain and worse function. Fatigue during the past week was assessed on the NRS from 0 (*no fatigue*) to 100 (*extreme fatigue*). To record the use of pain medication, participants were asked (yes/no) whether they used pain medication in the past 3 months for their hip or knee symptoms.

Patient knowledge of OA (treatments) was assessed using a self-administered questionnaire. Based on the frequently asked questions on OA reported in a previous study [26], 22 statements could be scored on a 4-point scale (*totally disagree* to *totally agree*, with the additional option *I don’t know*). A total score (maximum of 22) was calculated by awarding one point for each correct response. Each incorrect or undecided (*I don’t know*) answer was scored as 0.

Technology usage (frequency of internet and smartphone use in daily life) was assessed using 2 subscales of the Media and Technology Usage and Attitudes Scale (MTUAS) [27]. These scales use a 10-point frequency scale (1=never, 5=several times a week, 10=all the time) to score possible activities on a smartphone (eg, *using apps* or *listening to music*) and searching activities on the internet (eg, searching for information). Mean scores can be calculated for each subscale.

### Data Analysis

#### Descriptive Analysis

Baseline characteristics, user data, and usability were described descriptively using mean (SD), median (IQR), and number (%) where appropriate.

#### Statistical Analysis

The mean usability scores (SUS total scores and item scores) were compared between *superficial* and *active* users, using independent *t* tests (*P*<.05 for significance, two-tailed). The demographics and clinical characteristics were compared between *nonusers* and *users* and between *superficial* and *active users* of the educational eHealth tool using multivariable logistic regression analyses. First, the individual binominal associations between characteristics and the outcome variable (user or nonuser) were calculated. Variables with *P*≤.16 were selected for the multivariable logistic regression analyses [28]. By use of the Variance Inflation Factor (cutoff >10) statistic, the remaining variables were tested for collinearity [28]. A backward selection (*P*<.10 for removal) was used to generate the final model.

For the logistic regression analysis, multiple imputation using Imputation by Chained Equation was used to estimate the missing values. A total of 20 imputed datasets were combined using Rubin’s rules [29]. All data were analyzed using Stata 13.1.

## Results

### Participants

A total of 144 patients with hip or knee OA were included in this study. Their mean (SD) age was 61.7 (10.4) years, and more women (57%) participated than men. The mean (SD) BMI of the participants was 27.9 (4.4) kg/m^2^. A total of 57 (40%) participants had a low educational level (<12 years), and 58 (43%) had a paid job at the time of inclusion. The majority of participants had a scheduled consultation for a knee joint (80%). The duration of symptoms was less than 5 years for the majority of participants (58%). The participants had moderate-to-severe impaired functioning as reflected by the WOMAC (mean (SD): 50.6 (20.1) for pain and 55.1 (21.1) for functioning). Patient characteristics are shown in Table 1.

**Table 1:** Patient and clinical characteristics of users and nonusers of the educational electronic health tool.

Characteristics	Nonusers (n=28)	Users (n=116)
Gender (female), n (%)	14 (50.0)	67 (57.8)
Age (years), mean (SD)	59.4 (12.1)	62.2 (9.9)
BMI >25 kg/m^2^, n (%)	18 (64.3)	78 (67.2)
Married, n (%)	20 (71.4)	86 (74.1)
Level of education (>12 years), n (%)	17 (60.7)	67 (57.8)
Work status (paid), n (%)	11 (39.3)	47 (40.5)
Osteoarthritis of the knee, n (%)	21 (75.0)	94 (81.0)
Duration of symptoms (>5 years), n (%)	13 (48.1)	46 (39.7)
Pain, WOMAC^a^ (0-100), mean (SD)	58.1 (23.1)	49.1 (19.2)
Functioning, WOMAC (0-100), mean (SD)	62.4 (21.7)	53.5 (20.8)
Fatigue, NRS^b^ (0-100), mean (SD)	40.1 (20.2)	48.5 (25.1)
Pain medication use, n (%)	21 (75.0)	93 (80.2)
Knowledge of osteoarthritis^c^ (0-22), mean (SD)	9.7 (3.5)	11.4 (3.7)
Smartphone usage in daily life, MTUAS^d^ (1-10), mean (SD)	6.1 (1.7)	5.0 (1.9)
Internet usage in daily life, MTUAS (1-10), mean (SD)	6.3 (1.9)	4.8 (1.7)

^a^WOMAC: Western Ontario and McMaster Universities Osteoarthritis Index.

^b^NRS: numeric rating scale.

^c^Self-administered.

^d^MTUAS: Media and Technology Usage and Attitudes Scale.

### Use of the Educational Electronic Health Tool

**Figure 1:**
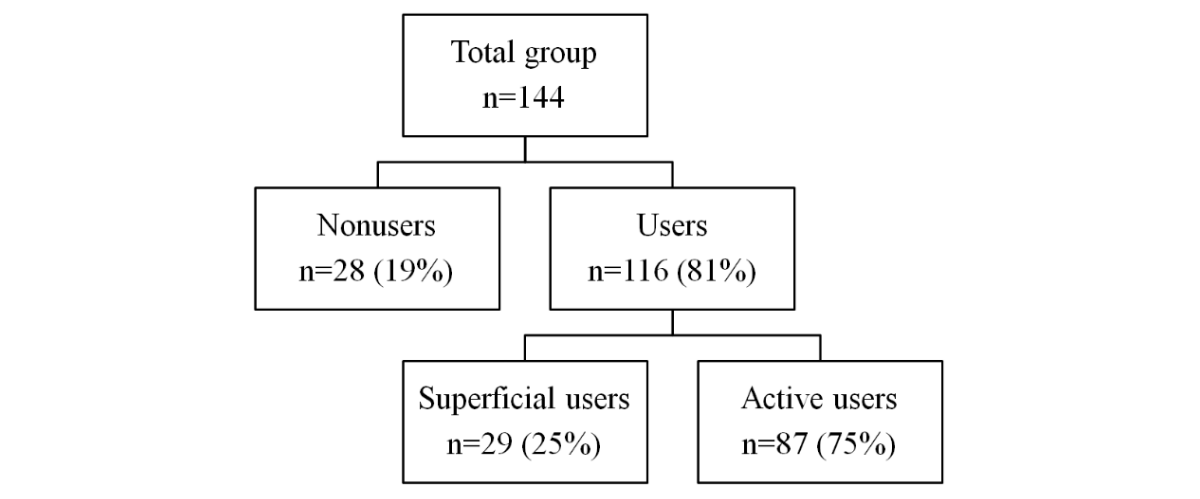
Distribution of nonusers and users (superficial and active) among the study population.

Among the participants, 116 (81%) were users and 28 (19%) were nonusers of the eHealth tool (Figure 1). The group of 116 users, comprised 29 (25%) superficial users and 87 (75%) active users.

Among the users, 74 (64%) participants used the Android or iOS app, and 57 (49%) used the Web version of the educational eHealth tool, with 15 participants using both platforms. The 3 main components of the tool (*information, my consultation, and medication*) were all opened by the majority of users (91%-95%; Table 2), whereas the short facts on OA were opened by slightly fewer users (80%). The median number of opened components was 2 (IQR 1-3). The actual use (ie, earning an achievement award) was highest for the preparation for the consultation (57%), followed by reading all the information on OA treatments (35%) and listing one’s medication (34%). A detailed look into the earned achievements revealed that participants who only earned one achievement received the award for the *information* or *my consultation* components. For those who earned 2 achievements, the combination of *my consultation* and *medication* was most frequently earned. Our results also show that of the 87 active users, 19 solely used the more passive component (ie, reading information), whereas more than half chose to use the interactive components alone (ie, preparing for a consultation only or in combination with listing medication).

**Table 2:** Use of components of the educational electronic health tool among 116 users.

Component		Participants, n (%)	Frequency, median (IQR)	Earned achievement, n (%)
**Information**				41 (35.3)^a^
	Opened *information*	110 (94.8)	4.5 (2-10)	—^b^
	Read ≥1 short fact	93 (80.2)	7.5 (1.5-11)	—
**My consultation**				66 (56.9)^c^
	Opened *my consultation*	109 (93.9)	6.5 (4-35)	—
	Answered ≥1 preparation question	63 (54.3)	—	—
	Recorded ≥ 1 questions for consultation	31 (26.7)	—	—
	Scheduled pain and/or fatigue measurements	38 (32.8)	—	—
**Medication**				40 (34.5)^d^
	Opened *medication*	105 (90.5)	6.5 (2.5-14.5)	—

^a^Visited all pages with information.

^b^Not applicable.

^c^Scheduled consultation date.

^d^Listed medication use.

### Questions Prepared for the Consultation

About one-fourth of users recorded one or more questions in preparation for their consultation. A total of 75 questions were recorded in the tool. Disagreement about the categorization of 20 questions was resolved by discussion. In all, 2 questions were excluded from the categorization because they were formulated as notes rather than questions. A total of 46 questions were categorized into the 3 predefined themes “What are my options?,” “What are the possible benefits and harms of those options?,” and “What does this mean in my situation?” (Table 3). When discussing the remaining 27 questions, the fourth theme was identified, “What is my situation at this moment?,” with 15 questions added to this category. The other 12 questions were grouped into the *remaining* category. These were mainly educational questions on how to deal with OA in daily life, some of which addressed the added value of experimental treatments.

**Table 3:** Categorization of questions prepared in the educational electronic health tool, with examples given for each theme.

Themes (number of questions)	Example questions
What is my situation at this moment? (15)	How far has the osteoarthritis progressed? What is the situation right now and what is the prognosis?
What are my options? (35)	What is your advice in resolving the pain? What are my treatment options?
What are the possible benefits and harms of those options? (5)	What can I expect if I had surgery? What is the recovery period of surgery?
What does this mean in my situation? (6)	Is it still necessary to use orthopedic shoes? Is it possible to get an injection in my knee one more time?
Remaining (12)	At what level can I be physically active with regard to the wear and tear of my cartilage? Is it possible to inject cartilage into the knee?

### Usability

The mean (SD) usability score among users, as measured with the SUS, was 64.8 (16.0). Moreover, patient satisfaction with the educational eHealth tool was 6.9 (1.7) on a scale from 0 to 10. No differences were found in the SUS and satisfaction scores between *active* and *superficial* users (mean difference 0.04, 95% CI −7.69 to 7.77 and mean difference 0.3, 95% CI −0.50 to 1.11, respectively). The comparison of individual items of the SUS between *active* and *superficial* users did also not result in any differences (results not shown).

### Subgroup Characteristics

Based on univariate binominal regression analyses, fatigue (*P*=.16), knowledge of OA (*P*=.04), and smartphone (*P*=.03) and internet (*P*=.009) use in daily life were included in a multivariable analysis. This analysis revealed that participants with a higher baseline knowledge of OA (OR 1.2, 95% CI 1.0 to 1.4) and who used the internet less frequently in their daily life (OR 0.6, 95% CI 0.5 to 0.9) were more likely to use the educational eHealth tool (Table 4). No statistically significant differences were found between the demographic and clinical characteristics of the superficial and active users.

**Table 4:** Results from the multivariable logistic regression analysis for differences between users and nonusers of the educational electronic health tool.

Variables	OR^a^ (95% CI)	*P* value
Knowledge of OA^b^ (0-22)	1.2 (1.0 to 1.4)	.02
Internet usage in daily life, MTUAS^c^ (1-10)	0.6 (0.5 to 0.9)	.003

^a^OR: Odds ratio.

^b^Self-administered.

^c^MTUAS: Media and Technology Usage and Attitudes Scale.

## Discussion

### Principal Findings

This study explored the use and usability of a smartphone and Web-based educational eHealth tool. The educational eHealth tool was used by 81% of the patients with knee or hip OA who were offered it. Among users, 75% actively engaged with the tool and used at least one of the components, with *information* and *my consultation* being the most popular components. Questions that were recorded by participants in preparation for their consultation were mostly in line with a widely used question prompt list, although a considerable number remained, some of which could be categorized in a new additional theme (“What is my situation at this moment?”). Participants with a higher baseline knowledge of OA and who used the internet less frequently in their daily life were most likely to use the tool. No other statistically significant differences were found between users and nonusers of the educational eHealth tool.

### Comparison With Previous Work

To our knowledge little is known about the use of stand-alone eHealth interventions in OA. Our finding that 81% of participants used the educational eHealth tool is in line with the results of De Vries et al, who evaluated adherence to a Web-based component of a blended care physical activity program for patients with hip or knee OA [30]. This study was part of a blended care intervention, involving interaction with a physiotherapist; therefore, it is not directly comparable with our study. One recently published RCT on the effectiveness of an educational smartphone and tablet app reported a 70% adherence rate [31]. Compared with other eHealth stand-alone interventions, these percentages are reasonably high [16,17]. The relatively high usage rate in our study could be explained by the short time frame in which the tool could be used (2 weeks before the upcoming consultation) and the specific objective of the tool.

In this study, we defined *users* and *nonusers* based on whether they opened the app. The users were defined as active when they opened the tool and earned at least one achievement award. In the development of the tool, all components included were indicated as important by stakeholders. However, we chose one achievement as cutoff for each individual user as there can be a difference in which components they want to use the app for. Using other cutoff points to define *use* might have resulted in different user rates and conclusions; however, considering the small differences we found and the lack of differences detected between the active and superficial users, it is not likely that changing the cutoff point would have resulted in additional findings of interest. However, this discussion does demonstrate the necessity of defining *use*. Currently, there is no consensus about how to define and appraise eHealth use; measures used to define use include the frequency of logging in or using a tool, the number of components used, or the time spent on the tool [32]. Often the threshold for *use* is drawn based on the concept that *more is better* or is not justified at all [33]. As every eHealth intervention has its own goal, it may not even be feasible or valuable to have one definition of *use*; however, we could strive for criteria that can be used to set a cutoff point for every intervention. This would help interpretation of eHealth *use* and facilitate comparisons between studies.

Regardless of the high user rates in our study, our results regarding usability and patient satisfaction about the tool show that there is still room for improvement. The mean usability score of our educational eHealth tool was 64.8 on a scale from 0 to 100, as measured with the SUS. Although this score corresponds to being fair to good [34], it does not reach the acceptable score (ie, 70) proposed by Bangor et al [35]. Previous studies on the usability of eHealth and mobile health (mHealth) tools had considerably higher SUS scores [36,37]. Scott et al reported a median (IQR) SUS score of 95 (86-98) immediately after providing instructions about a mobile app for daily postoperative self-reporting after colorectal surgery [36]; however, the majority of participants did not use the app after discharge or only used it once. This indicates that high usability alone is not sufficient to motivate people to use eHealth tools [36].

The Technology Acceptance Model states that the actual use of a technology system is determined by both the perceived usefulness (utility) and the perceived ease of use (usability) [38]. We found that the eHealth tool was used by a fair number of participants, despite the fact that its usability was somewhat disappointingly rated by users. This may indicate that patients see the benefits of using the educational eHealth tool in preparation for their consultation (utility). It is therefore conceivable that improvement of the content might increase the usability and ultimately optimize patient motivation to use the eHealth tool. Frie et al evaluated reviews of smartphone app for monitoring weight loss and found that users had a preference for apps with a limited number of features [39]. Our eHealth tool contained 3 components (*information, my consultation,* and *medication*), each of which contained multiple features (eg, monitoring pain and fatigue, recording questions, and answering predefined questions). For further improvement of the tool, the removal of the *medication* component should be considered, as this component was the least used by participants. Remarkably, the component *medication* was added on the request of the end users during the iterative development process; however, it is possible that we made the tool too complicated by taking too many requests into account.

An important part of our intervention was the preparation for the consultation, which involved listing questions to ask during the consultation. Currently, 3 standardized questions (“What are my options?,” “What are the possible benefits and harms of those options?,” and “How likely are the benefits and harms of each option to occur?”) are used in several national campaigns in England, Australia, and the Netherlands [12]. However, it is not known to what extent these questions cover the essence of the questions patients want to ask. Here, we compared the listed questions with the 3 standardized questions. Our results showed that about a quarter of the questions listed by the participants did not fit these 3 themes. A considerable number of these remaining questions were focused on the current status/situation that patients were in, for example, “how far has my OA progressed?” and “what is the prognosis based on my current situation?” This shows that although prompting predefined questions may result in patients considering novel topics [40], it may also miss patient’s individual information needs. This consideration is in line with a recent RCT performed by Bottachini et al, who compared the use of a question prompt list (predefined questions) with a question list in breast cancer patients and found that patients who used the prompt list were less satisfied with the information they received during their consultation [40]. Our results support the extension of the 3 standardized questions to 4, but also suggest the importance of finding ways to elicit the individual information needs of patients not covered in the predefined questions to optimize their preparation for consultations, for instance, by providing a space for a list of their own questions, as we did in our educational eHealth tool.

We found several differences between the characteristics of users and nonusers. Our results show that the baseline knowledge of OA was lower among nonusers than users, suggesting that some subgroups of patients may just not be as interested in learning more about their condition or are not able to [41]. In clinical practice, it is important to be aware of this subgroup of patients, which may need a different strategy to be educated. In addition, we found that users were less familiar with using the internet in their daily life than nonusers. Although only univariate, the same trend was seen for daily life smartphone usage (*P*=.03). A previous study on the determinants of adherence to a Web-based component of a physical activity program in OA qualitatively identified internet skills as important for optimal adherence [30]. On average, the users in our study indicated that they use the internet and smartphones several times a week, which suggests that they likely had significant skills in using these media. The literature also shows that younger people are less likely to adhere to eHealth tools [16,42]. Although not statistically significant, the nonusers in our study were slightly younger than the users. It is likely that younger adults, who in general use the internet and smartphones more often [43], have lost interest in new apps that are continuously being offered to them or already found similar apps or information about OA on the internet. Different strategies to target this subgroup should be explored.

### Strengths and Limitations

Several limitations of this study need to be addressed. First, we do not know why some participants did not use the app or only used it in a superficial way. Qualitative research may provide additional insights into how we can further optimize the eHealth app. Second, we used the SUS questionnaire to obtain information on the usability of the app at one point in time. The SUS has been shown to be reliable and is the most widely used questionnaire for evaluating usability, making it easy to compare with other eHealth interventions [44]. Despite these benefits, the SUS was not originally developed with a focus on health care and therefore only provided us with a general idea of the tool’s usability. Comparing the SUS scores with more objective outcomes like effectiveness and efficiency could be of added value in this regard. Also, measuring the SUS at more points in time, before and after the previously suggested adjustments to the tool, would provide a more specific picture of the tool’s usability. Finally, it is important to note that our study sample consisted of patients willing to participate in an RCT evaluating an eHealth app. Although patients who were not willing to participate did not differ from those who did in terms of their age or gender, we do not know the extent of internet use by the patients not willing to participate; therefore, it is not possible to generalize the user characteristics we identified to the entire OA population. To consolidate our results in future research, we recommend the use of a study design in which every patient visiting for the first orthopedic consultation gets access to the educational eHealth tool.

### Conclusions

On the basis of relatively high user rates and reasonable usability scores, it can be concluded that the use of an educational eHealth tool to prepare patients with hip and knee OA for their first orthopedic consultation is feasible; however, improvements to the content of the tool itself should be established to enhance its usability and user satisfaction. It is recommended that 44 predefined questions as mentioned in this study are included and that space is provided for patients to list additional questions to support their preparation for their consultation. Moreover, simplifying the tool may also increase its usability. We found no clear practical indications that specific subgroups should be targeted for implementation. The literature on the use of eHealth and especially mHealth technologies in patients with OA is scarce. The results of this study therefore provide new insights revealing that interventions using eHealth have the potential to reach this population and show that usage data can reveal how to further optimize the delivery of these interventions.

## Appendix

Multimedia Appendix 1 Development and functionalities of the educational eHealth tool.

## Abbreviations

BMI: body mass index
eHealth: electronic health
mHealth: mobile health
MTUAS: Media and Technology Usage and Attitudes Scale
OA: osteoarthritis
OR: odds ratio
RCT: randomized controlled trial
SUS: System Usability Scale
TJR: total joint replacement
WOMAC: Western Ontario and McMaster Universities osteoarthritis index
