# Supplementary material for: An Electronic Health Tool to Prepare for the First Orthopedic Consultation: Use and Usability Study
Source: JMIR Form Res. 2019 Nov 28;3(4):e13577. doi: 10.2196/13577 (PMC6913511; doi:10.2196/13577)
Supplement: Multimedia Appendix 1 [file formative_v3i4e13577_app1.pdf]

Multimedia Appendix 1 "Development and functionalities of the educational eHealth tool"

Development

Based on patient responses in daily practice to a previous implemented self-management booklet we choose to develop the educational eHealth tool [1]. We followed an eight-step method of persuasive design [2]. Persuasive technology is the use of technology (internet, mobile devices like smartphones, tablets, e-mail) to change people's behavior and influence their choices. Research shows that online applications, which are developed by using the methodology of persuasive design, are leading to better treatment adherence [3]. A project group consisting of a project leader with expertise in persuasive design, a senior researcher, 4 students and 4 patients with osteoarthritis (OA) were responsible for the execution of the different steps. First, by means of individual interviews with a feedback group of eight patients with OA information needs regarding 1) a previously developed multidisciplinary, guideline-based stepped-care strategy for patients with knee and hip osteoarthritis and health care providers [4] and 2) preferences and barriers regarding the use of technology were examined. Main results regarding needs were "tailored information", "assistance in preparing consultations with care providers", "need for tool to log important health information related to osteoarthritis" and "need for tool to log appointments with care provider". Next, based on the results of the interviews the primary goals of the tool were defined after discussion with all members of the project group. Those goals were translated to "actual target behaviors", e.g. "start internet application", "register for account"; "click for more detailed information" and "log questions for care provider". Then, for each target behavior a paper prototype was produced consisting of the different "screens" by the student members of the project group. These paper prototypes were iteratively tested on feasibility and clarity by the members of the patient feedback group, utilizing the "speaking aloud" method and adapted several times [5]. The paper version was used to specify functionalities of the educational eHealth tool.

Based on specified functionalities a software development company (Comaxx – e-business) started the development of the tool using the project management method Scrum. The Scrum method is an iterative process in which the tool was developed in 4 sprints of 4-6 weeks. Each sprint followed the phases of development, user-testing, adaptation, re-testing and finalizing. In each user-testing phase a group of 5-10 patients and health care providers gave their feedback on the educational eHealth tool.

Functionalities

The final version of the educational eHealth tool as used in the study was available as native application for iOS and Android (Version 1.1, release date February 14th 2017), and as a web based version (www.artrosewijzer.nl) free of charge. The tool can only be accessed with an username and password, provided by the researcher. The educational eHealth tool has the following parts:

1. “Information” (Figure 1). Information on OA and treatment modalities, based on a stepped-care strategy for OA [4], with additional information on surgical treatment.
2. “My consultation” (Figure 1). In this part the user gets the option to record a consultation by filling in date, time, location, healthcare professional and subject. Based on the type of healthcare professional (orthopedic surgeon, rheumatologist, physiotherapist, other) questions can be answered that likely will be asked during the consultation. Additionally, users can record questions they want to ask themselves during the consultation. During the week before the upcoming consultation it is possible to daily monitor fatigue or pain on a numeric rating scale (0 to 10). Users receive a notification at the time they planned a measurement.
3. “Medication” (Figure 2). The possibility to list medication use (e.g. dosage) with the option to set reminders for intake
4. Achievements (Figure 2). It is possible to earn three achievements while using the educational eHealth tool. The first one can be earned when users read all the information

parts in the tool. The second one can be earned when a consultation is planned. The last one is received when medication use is registered in the app.

5. Timeline (Figure 3). Based on a planned consultation a visual timeline is created in the tool, along with measurement moments and preparation. One day before the consultation the user receives a notification to prepare the consultation.

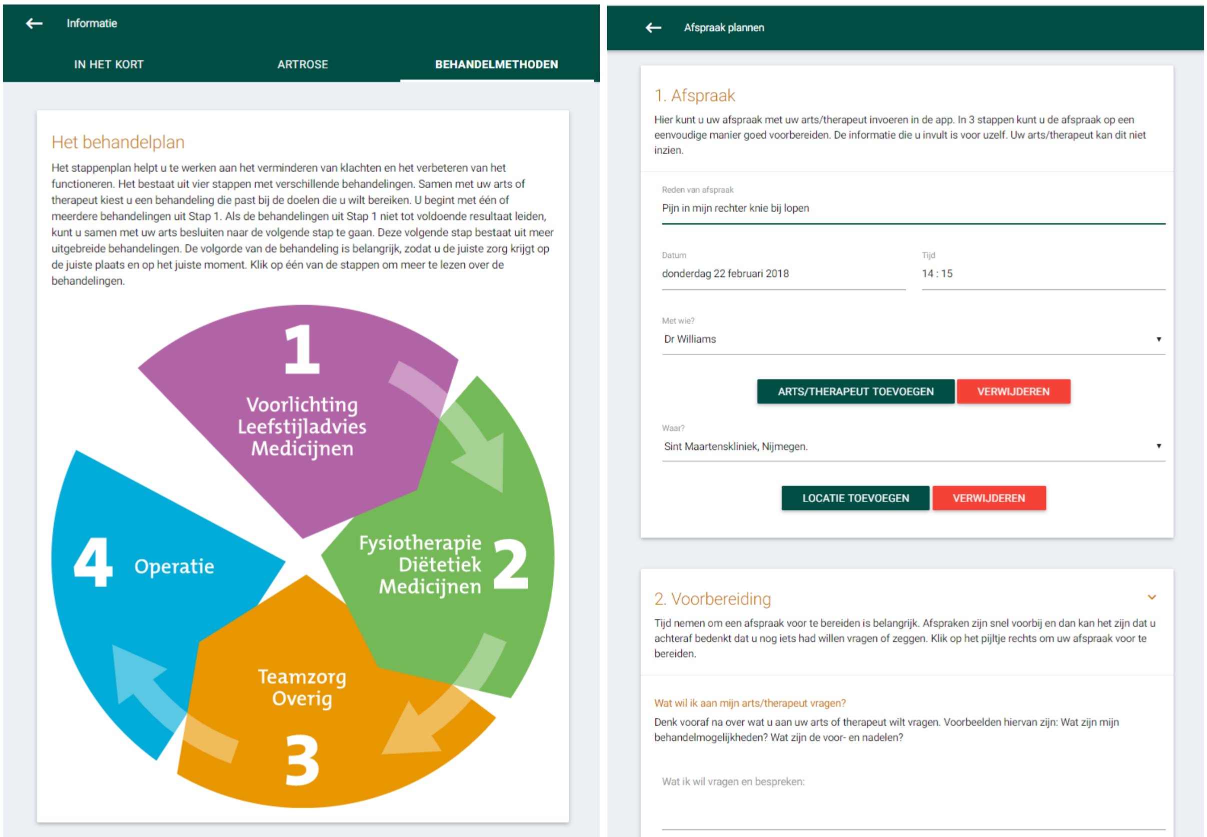

Figure 1 Screenshots of the stepped care strategy, providing information on treatment options for hip and knee OA (left) and consultation preparation (right) in the educational eHealth tool.

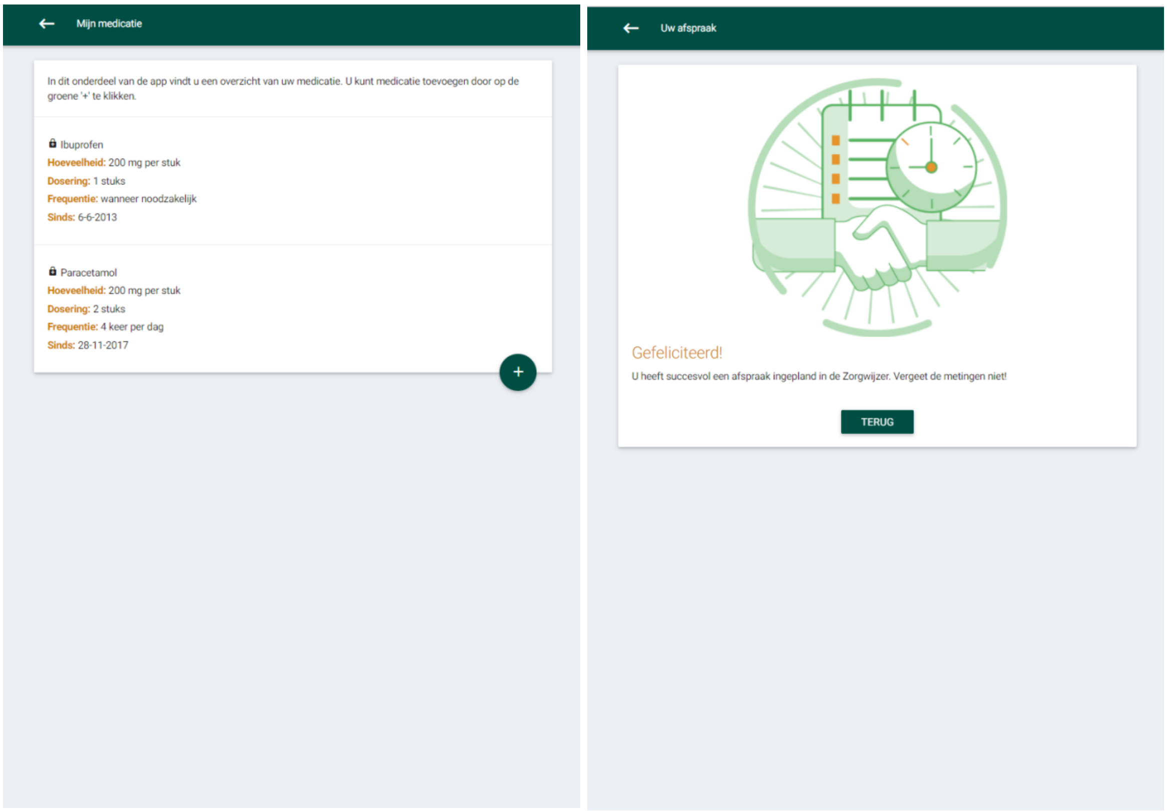

Figure 2 Screenshots of medication listed (left) and an earned achievement for recording a consultation.

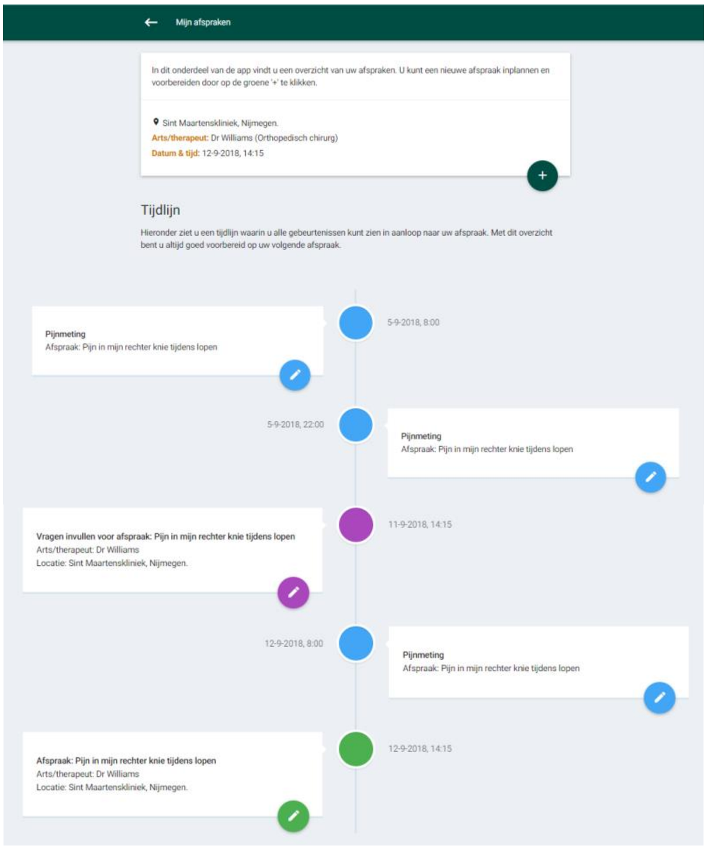

Figure 3 Screenshot of a created timeline with a planned consultation, scheduled measurement moments and preparation.

Reference List

1. Cuperus N, Smink AJ, Bierma-Zeinstra SM, Dekker J, Schers HJ, et al. Patient reported barriers and facilitators to using a self-management booklet for hip and knee osteoarthritis in primary care: results of a qualitative interview study. BMC Fam Pract 2013; 14:181.
2. Fogg BJ. Creating persuasive technologies: an eigth-step design process. Proceedings of the 4th International Conference on Persuasive Technology. 2009: 1-6.
3. Kelders SM, Kok RN, Ossebaard HC, Van Gemert-Pijnen JE. Persuasive system design does matter: a systematic review of adherence to web-based interventions. J Med Internet Res 2012; 14(6):e152.
4. Smink AJ, van den Ende CH, Vliet Vlieland TP, Swierstra BA, Kortland JH, Bijlsma JW et al. "Beating osteoARThritis": development of a stepped care strategy to optimize utilization and timing of non-surgical treatment modalities for patients with hip or knee osteoarthritis. Clin Rheumatol 2011; 30(12):1623-9.
5. van Someren MW, Barnard Y, Sandberg J. The thinking aloud method: a practical approach to modelling cognitive processes. Londen: Academic Press; 1994.
